# Health literacy affected the residents’ knowledge, attitude, practice for prevention and control of COVID-19 in Shanxi Province, China

**DOI:** 10.1038/s41598-023-30730-9

**Published:** 2023-03-02

**Authors:** Jianchun Ling, Hui Jiang, Xuchun Wang, Huaxiang Rao

**Affiliations:** 1 Department of the Second Editorial, Comprehensive Service Center of Shanxi Medical and Health Institutions (Shanxi Province Blood Center), Taiyuan, 030013 Shanxi China; 2 Department of Computer Teaching, Changzhi Medical College, Changzhi, 046000 Shanxi China; 3 Department of Health Statistics, School of Public Health, Shanxi Medical University, Taiyuan, 030001 Shanxi China; 4 Department of Public Health and Preventive Medicine, Changzhi Medical College, Changzhi, 046000 Shanxi China

**Keywords:** Diseases, Risk factors

## Abstract

Multistage stratified random sampling was used to explore the relationship of health literacy with novel coronavirus disease 2019 (COVID-19) prevention and control knowledge, attitude and practice (KAP) in residents aged 15–69 years old in Shanxi Province. The questionnaire, which was issued by the Chinese Center for Health Education, consisted of a health literacy questionnaire and a COVID-19 prevention and control KAP questionnaire. According to the national unified scoring method, the participants were divided into two groups: those who with adequate health literacy and those who with inadequate health literacy. The results of the answer to each KAP question were compared between the two groups by Chi-square test or Wilcoxon rank sum test. Binary logistic regression was used to control confounding effects of socio-demographic characteristics to draw relatively reliable conclusions. A total of 2700 questionnaires were distributed, and 2686 valid questionnaires were returned, with an efficiency rate of 99.5%. Health literacy qualified was identified for 18.32% (492/2686) in Shanxi Province. Compared with the inadequate health literacy group, people with adequate health literacy had a higher corrected answer rate in 11 knowledge-related questions (all *P* < 0.001); showed more positive answer to each attitude-related question in the three aspects, namely, responsibility for the prevention and control of infectious disease transmission, evaluation for COVID-19-related information release and reporting, and evaluation for the government’s COVID-19 prevention and control results (all *P* < 0.001); and acted more actively in the practice concerning appropriate self-prevention and control behaviors during the COVID-19 outbreak (all *P* < 0.001). Logistic regression analyses confirmed that with adequate health literacy played a positive role in each of the contents of COVID-19 prevention and control KAP (ORs were between 1.475 and 4.862, all *P* < 0.001). Health literacy is closely related to COVID-19 prevention and control KAP in the general population of Shanxi Province. People with high score of health literacy were generally better able to grasp COVID-19 prevention and control knowledge, have more positive attitudes toward prevention and control, and perform better prevention and control behaviors. Promoting residents’ health literacy by targeted health education can play an important and positive role in dealing with the threat of major infectious diseases outbreaks.

## Introduction

The novel coronavirus disease 2019 (COVID-19) was first reported in Wuhan, Hubei Province, China, in the end of December 2019^1^. Subsequently, the World Health Organization (WHO) named the novel coronavirus disease 2019 “COVID-19” on February 11, 2020^2^. Since COVID-19 is a recently developing infectious disease that is extremely contagious, spreads swiftly, and advances quickly in terms of disease, humans are not immune to it. The pandemic had spread rapidly across the World and has caused heavy burden and severe challenges to society and economy and has brought tremendous threats to human life^3,4^. Since the spread of the epidemic, various countries have responded with measures to control the further development of the epidemic. A range of antiviral vaccines for COVID-19 have emerged. WHO recommends vaccination as the most appropriate method to establish herd immunity in a population^5^, and the vaccines available for administration to the population have been reported to be safe, they have been associated with deaths due to allergic reactions and thrombotic events only in rare cases^6^. And vaccination has been effective in reducing and mitigating the spread of some outbreaks and the severity of infection symptoms, while greatly reducing the disease mortality rate. However, in the presence of these unwanted adverse events after vaccination and the lack of clear information about the duration of efficacy, many people still have doubts about the COVID-19 vaccine^7^. Results of a rapid systematic review and meta-analysis study of a large, nationally representative sample of intended uptake and refusal of the COVID-19 vaccine indicate that as the pandemic has progressed, the percentage of people intending to vaccinate decreased and the percentage of people intending to refuse vaccination increased^8^. A recent global study also showed that only 71.5% of people were willing to be vaccinated with COVID-19, with the Chinese having the highest willingness (88.6%) and the Russians having the lowest willingness (54.9%)^9^. In an earlier study, it was observed that 33% of US respondents showed hesitation to receive COVID-19 vaccine^10^. Similarly, in an online survey, about 31% of Turkish participants reported refusal of COVID-19 vaccination^11^. In general, the importance of the vaccine and its preventive effect on COVID-19 has received a relatively positive response in many studies, yet there is still a proportion of people who are reluctant or hesitant to be vaccinated. This also affects the prevention and control of the epidemic to some extent. Besides, the high variability of the virus, with multiple mutant strains of new coronaviruses having emerged since 2020 to date, has also greatly increased the difficulty of preventing and combating the epidemic. In addition, at present, COVID-19 is mainly based on symptomatic and supportive treatment^12^. Therefore, prevention and control should be focus to combat the pandemic^13^. While people’s personal behaviors, like compliance with maintaining physical distance, voluntary testing, self-isolation and hand hygiene were the effective measure to prevent and control the spread of infectious diseases like COVID-19. Some of these behaviors can be reinforced by a range of restrictions introduced by the state and government, such as travel restrictions, requiring self-isolation for returning travelers, closure of public entertainment venues, significant restrictions on personal contact, and requirements to work or attend school remotely^14,15^. The domestic prevention and control policy has changed significantly in light of the current deregulated environment in China. Positive infected and confirmed patients are gradually no longer isolated, and check travel codes, nucleic acid testing, and other measures are gradually discontinued. A large number of infections have spread quickly across the country. However, the prevention and control measures and recommendations for the people are still to keep a distance, wear masks, etc.. Moreover, due to the liberalization of national control measures, the importance of personal prevention and control has been expanded. Residents with good health literacy and good knowledge, positive attitudes, acceptable practices, and good Health literacy for COVID-19 are more likely to be able to reduce the chance of infection, or repeat infection, and those with underlying diseases should pay more attention to personal protection. On the other hand, personal health literacy and awareness of COVID-19 are even more important in the current environmental context. However, because different levels of individual engagement with the above restrictions may lead to hot spots or additional waves of infection, and certain groups may be more severely affected by COVID-19, an effective response to the virus will also require individuals to change their behavior. This means that people must be able to process and understand rapidly evolving public health information and then act on it.

People are known to have different abilities to understand, access and act on health advice and make informed health decisions, a set of skills commonly referred to as “health literacy”. Health literacy is the ability of individuals to access and understand health information knowledge and related services, and to manage their own health through this information^16^. People with low health literacy may not have access to effective health knowledge and implement good health behaviors^17^. Previous studies have also found that increasing health literacy levels can effectively help residents prevent and control disease^18–20^. This suggests that health literacy is a key factor in managing the COVID-19 outbreak in the face of a sudden major infectious disease outbreak^21^, a 100-year outbreak of New Crown Pneumonia, and provides a new perspective for future health literacy research in the context of viral outbreaks^22^. A few studies have analyzed the relationship between health literacy and KAP for prevention and control of COVID-19, e.g., Kirsten J McCaffery et al. conducted a nationwide cross-sectional survey study of the Australian public aged 18 years and older in 2020. The study analyzed the variation in understanding of, attitudes towards, and uptake of, health advice on coronavirus disease 2019 (COVID-19) during the 2020 pandemic stage 3 restrictions (‘lockdown’) by health literacy in the Australian population. The findings showed that there are important disparities in COVID-19 related knowledge, attitudes and behaviours according to peoples health literacy and language^12^. However, the study was limited to Australia, and in China, the article on COVID-19 was limited to the Baoji. The results of the study showed that the health literacy is closely related to COVID-19 prevention and control KAP in the general population of Baoji city^23^. Although a nationally representative sample was not obtained for this study, it was still able to provide some insight into the health literacy of Shanxi Province residents and to demonstrate the relationship between health literacy and KAP for COVID-19 among Shanxi Province residents, further highlighting the significance of residents’ health literacy on their knowledge, attitudes, and behaviors regarding COVID-19 prevention. It also compensates for the paucity of national studies on this subject in China and contributes some important data to regional studies on the subject there. Besides, understanding this relationship will not only enhance the community’s awareness of the importance of residents’ health literacy, but also facilitate the prevention and control of outbreaks of major infectious diseases for the whole population.

The purpose of this study is to investigate the relationship between health literacy and KAP for prevention and control of COVID-19 by taking advantage of the timing of the National Health Literacy Surveillance Current Survey in September–November 2020 to aggregate and analyze relevant data from residents of Shanxi Province. To understand how people understand and interpret health information at critical moments, and to explore the link between population health literacy level and KAP for prevention and control of COVID-19, in order to provide scientific basis for improving the health literacy of the whole population and resisting the threat of sudden major infectious disease epidemics.

## Results

### Sociodemographic characteristics

A total of 2700 questionnaires were distributed, and 2686 residents filled out the questionnaire, with an effective response rate of 99.5% (2686/2700). All subjects were not infected with the virus of COVID-19 and were not close contacts. The general characteristics of the study population are presented in Table 1. Among the 2868 participants, 43.66% were male and 56.34% were female. The participants were divided into three groups based on their ages: 15–44, 45–59, and 60–69. The majority of participants were married (83.95%), and had a junior high school education (45.49%). In total, 18.32% (492/2686) of the participants were with adequate health literacy. Significant differences were found in age, education level, per capita annual income and occupation between the adequate and inadequate health literacy groups (*P* < 0.05).

**Table 1:** Basic characteristics of respondents and association with health literacy.

Characteristics		Total	Inadequate health literacy (*n* = 2194)		Adequate health literacy (*n* = 492)		*χ*^2^	*P*
			Number	%	Number	%		
Gender	Male	1173	958	81.67	215	18.33	< 0.01	0.989
	Female	1513	1236	81.69	277	18.31		
Age	15–44	792	602	76.01	190	23.99	33.64	< 0.001
	45–59	1126	921	81.79	205	18.21		
	60–69	768	671	87.37	97	12.63		
Education level	Primary school and below	671	611	91.06	60	8.94	183.07	< 0.001
	Junior high school	1163	998	85.81	165	14.19		
	Senior high school and technical	502	377	75.10	125	24.90		
	College and above	350	208	59.43	142	40.57		
Per capita annual income	≤ ¥5000	958	802	83.72	156	16.28	15.58	< 0.001
	¥5001–¥14,999	829	695	83.84	134	16.16		
	≥ ¥15,000	899	697	77.53	202	22.47		
Occupation	Farmers	1543	1343	87.04	200	12.96	69.51	< 0.001
	Others	1143	851	74.45	292	25.55		
Marriage status	In marriage	2241	1820	81.21	421	18.79	1.99	0.158
	Others	445	374	84.04	71	15.96		
Total	–	2686	2194	81.68	492	18.32	–	–

### Health literacy of respondents and association with COVID-19-related prevention and control knowledge

Table 2 demonstrates 11 questions assessing knowledge (A) of participants regarding the COVID-19 prevention and control during the pandemic. The overall knowledge of COVID-19 prevention and control was poor among the 2686 respondents. The highest correct response rate was 92.26% (2478/2686) for “A2: Number of days of medical observation in isolation for close contacts”; The percentages of correct answers for “A10: Proper practice during group meals”, “A3: COVID-19 susceptible individuals”, and “A7: Correct way to wash hands” were more than 50%, in the following order 74.83% (2010/2686), 57.68% (1549/2686), 50.56% (1358/2686). The correct response rates for “A4: Sources of COVID-19 transmission”, “A5: Routes of COVID-19 transmission”, and “A6: Protective measures against COVID-19” were low, at 20.22% (543/2686), 9.57% (257/2686), and 18.17% (488/2686), respectively. Moreover, those who with adequate health literacy had a higher percentage of correct answers in all the above 11 questions than those who with inadequate health literacy, and the difference was statistically significant (all *P* < 0.001).

**Table 2:** Correct answer rates of COVID-19 prevention and control knowledge questions in the residents who with adequate health literacy and with inadequate health literacy, respectively.

Numbers of correct answer		Inadequate health literacy (*n* = 2194)		Adequate health literacy (*n* = 492)		*χ*^2^	*P*
		Correct number	Correct rate (%)	Correct number	Correct rate (%)		
A1	929	696	31.72	233	47.36	43.42	< 0.001
A2	2478	1990	90.70	488	99.19	40.50	< 0.001
A3	1549	1180	53.78	369	75.00	74.11	< 0.001
A4	543	417	19.01	126	25.61	10.87	0.001
A5	257	186	8.48	71	14.43	16.46	< 0.001
A6	488	316	14.40	172	34.96	114.22	< 0.001
A7	1358	968	44.12	390	79.27	198.61	< 0.001
A8	1083	808	36.83	275	55.89	60.71	< 0.001
A9	715	430	19.60	285	57.93	302.24	< 0.001
A10	2010	1528	69.64	482	97.97	171.18	< 0.001
A11	1205	828	37.74	377	76.63	245.68	< 0.001

A1: How many degrees above the body temperature need to seek medical attention; A2: Number of days of medical observation in isolation for close contacts; A3: COVID-19 susceptible individuals; A4: Sources of COVID-19 transmission; A5: Routes of COVID-19 transmission; A6: Protective measures against COVID-19; A7: correct way to wash hands; A8: What kind of mask is preferred; A9: Single-use medical mask wearing method; A10: Proper practice during group meals; A11: Personal protective measures for resumption of work and production in low-risk areas

### Health literacy of respondents and association with the COVID-19-related prevention and control attitude

Table 3 elucidates six questions assessing the attitude (EA) of participants regarding the responsibility of citizens during epidemics of infectious diseases. The overall attitude of the 2686 respondents towards the responsibility of citizens in the epidemic of infectious diseases is good, in descending order: (1) EA2: Cooperate with flow investigation and sampling (83.99%, 2256/2686); (2) EA3: Cooperate with isolation and observation, treatment, etc. (80.49%, 2162/2686); (3) EA1: Promptly report infectious patients or suspects (77.40%, 2079/ 2686); (4) EA4: Do not buy and eat wild animals (69.40%, 1864/2686); (5) EA5: Do not create rumors or inflate prices (63.22%, 1698/2686); (6) EA6: Do not discriminate against infectious patients, suspects or pathogen carriers (54.13%, 1454/2686). The attitudes of those with adequate health literacy were higher than those with inadequate health literacy in all six areas, and the differences were statistically significant (all *P* < 0.001).

**Table 3:** Results of answers to COVID-19 prevention and control attitude-related questions in the evaluation for COVID-19-related information release and reporting in the residents who with adequate health literacy and with inadequate health literacy, respectively.

Numbers of “yes” answer		Inadequate health literacy (n = 2194)		Adequate health literacy (n = 492)		*χ*^2^	*P*
		yes	%	yes	%		
EA1	2079	1612	73.47	467	94.92	105.67	< 0.001
EA2	2256	1781	81.18	475	96.54	70.61	< 0.001
EA3	2162	1692	77.12	470	95.53	86.73	< 0.001
EA4	1864	1409	64.22	455	92.48	151.11	< 0.001
EA5	1698	1262	57.52	436	88.62	167.13	< 0.001
EA6	1454	1046	47.68	408	82.93	201.13	< 0.001

EA1: Promptly report infectious patients or suspects; EA2: Cooperate with flow investigation and sampling; EA3: Cooperate with isolation and observation, treatment, etc.; EA4: Do not buy and eat wild animals; EA5: Do not create rumors or inflate prices; EA6: Do not discriminate against infectious patients, suspects or pathogen carriers.

Table 4 reveals 6 questions assessing the evaluations (EB/EC) of participants regarding the COVID-19 related information reports and the effectiveness of government prevention and control during the pandemic. Respondents’ agreement with the release and coverage of COVID-19 related information (EB1-5) was around 95% (93.18–96.49%). The proportion of respondents who rated the effectiveness of government prevention and control as good or very good was 96.02% (2579/2686). The approval rate of information reporting and the recognition of government prevention and control effectiveness were higher among those with adequate health literacy than those with inadequate health literacy, and the differences were statistically significant (all* P* < 0.001).

**Table 4:** Results of answers to COVID-19 prevention and control attitude-related questions in the evaluation for the government’s COVID-19 prevention and control results in the residents who with adequate health literacy and with inadequate health literacy, respectively.

Question		Total (n = 2686)		Inadequate health literacy (n = 2194)		Adequate health literacy (n = 492)		*Z*	*P*
		num	%	num	%	num	%		
EB1	Very dissatisfied	9	0.34	9	0.41	0	0.00	− 5.36	< 0.001
	Not satisfied	12	0.45	12	0.55	0	0.00		
	Basically satisfied	73	2.72	70	3.19	3	0.61		
	Satisfaction	690	25.69	596	27.16	94	19.11		
	Very satisfied	1902	70.81	1507	68.69	395	80.28		
EB2	Very dissatisfied	8	0.30	8	0.36	0	0.00	− 6.60	< 0.001
	Not satisfied	7	0.26	7	0.32	0	0.00		
	Basically satisfied	88	3.28	86	3.92	2	0.41		
	Satisfaction	968	36.04	835	38.06	133	27.03		
	Very satisfied	1615	60.13	1258	57.34	357	72.56		
EB3	Very dissatisfied	5	0.20	5	0.20	0	0.00	− 6.24	< 0.001
	Not satisfied	9	0.30	9	0.40	0	0.00		
	Basically satisfied	116	4.30	112	5.10	4	0.80		
	Satisfaction	940	35.00	805	36.70	135	27.40		
	Very satisfied	1616	60.20	1263	57.60	353	71.70		
EB4	Very dissatisfied	7	0.26	7	0.32	0	0.00	− 6.56	< 0.001
	Not satisfied	14	0.52	14	0.64	0	0.00		
	Basically satisfied	131	4.88	123	5.61	8	1.63		
	Satisfaction	1001	37.27	859	39.15	142	28.86		
	Very satisfied	1533	57.07	1191	54.28	342	69.51		
EB5	Very dissatisfied	23	0.86	20	0.91	3	0.61	− 5.73	< 0.001
	Not satisfied	16	0.60	16	0.73	0	0.00		
	Basically satisfied	144	5.36	133	6.06	11	2.24		
	Satisfaction	1085	40.39	920	41.93	165	33.54		
	Very satisfied	1418	52.79	1105	50.36	313	63.62		
EC	Poor	6	0.22	6	0.27	0	0	− 5.11	< 0.001
	Relatively poor	14	0.52	14	0.64	0	0		
	Fair	87	3.24	83	3.78	4	0.81		
	Good	567	21.11	490	22.33	77	15.65		
	Very good	2012	74.91	1601	72.97	411	83.54		

EB1: Timely release of information, open and transparent; EB2: Information source Scientific authority; EB3: Sufficient information to meet needs; EB4: The presentation of information is easy to understand; EB5: The information content is practical and the behavior is instructive; EC: How effective is the government's prevention and control.

### Health literacy of respondents and association with the COVID-19-related prevention and control practice

Table 5 reveals seven questions assessing the practice (B1-7) of participants regarding the practice towards COVID-19 during the pandemic. Majority of the respondents had a good practice towards the COVID-19 pandemic. More than half of the respondents had three aspects of COVID-19 outbreak prevention and control behaviors, namely, “B1: Actively searching for prevention and control knowledge”, “B4: Consulting community doctors by phone or on site”, and “B5: Actively inquiring about the outbreak near their place of residence”. While the behavior of actively seeking psychological or phone consultation was less, such as “B2: Call 12,320 for consultation (31.98%, 859/2686)”, “B3: Call psychological hotline (28.18%, 757/2686)”, “B6: Actively inquiring information about the same ride (29.90%, 757/2686)”, and “B7: Consulting a doctor online on the Internet (19.14%, 514/2686)”. Those with adequate health literacy had higher rates of practices in B1, B3, B5, B6 and B7 than those with inadequate health literacy (all *P* < 0.05).

**Table 5:** Rates of appropriate behaviors during the COVID-19 outbreak in the residents who with adequate health literacy and with inadequate health literacy, respectively.

Behavior	Total		Inadequate health literacy (n = 2194)		Adequate health literacy (n = 492)		*χ*^2^	*P*
	Yes	%	Yes	%	Yes	%		
B1	1584	58.97	1211	55.20	373	75.81	70.60	< 0.001
B2	859	31.98	706	32.18	153	31.10	0.22	0.642
B3	757	28.18	641	29.22	116	23.58	6.31	0.012
B4	1528	56.89	1229	56.02	299	60.77	3.71	0.054
B5	1382	51.45	1026	46.76	356	72.36	105.39	< 0.001
B6	803	29.90	602	27.44	201	40.85	34.51	< 0.001
B7	514	19.14	396	18.05	118	23.98	9.15	0.002

B1: actively searching for prevention and control knowledge; B2: call 12,320 for consultation; B3: call psychological hotline; B4: call psychological hotline; B5: actively inquiring about the outbreak near their place of residence; B6: actively inquiring information about the same ride; B7: online consultation with doctors on the Internet.

### Multiple unconditional binary logistic regression model for identification of the determinants significantly associated with COVID-19-related prevention and control KAP

In this study, knowledge of prevention and control, responsibility of citizens to prevent and control infectious disease epidemics, evaluation of information reports related to COVID-19, evaluation of the effectiveness of government prevention and control, and prevention and control behaviors were used as dependent variables, and sociological demographic characteristics, health literacy and its three aspects and six dimensions were assigned as independent variables, respectively (Table 6), and multifactorial unconditional binary logistic regression analysis was performed.

**Table 6:** Variable assignment table.

Variables	Assignment	Variables	Assignment
Knowledge of prevention and control	1 = ≤ 5 score, 2 = > 5 score	Health literacy	1 = Qualified, 0 = Unqualified
Responsibility of citizens to prevent and control the epidemic	1 = < 5 score, 2 = ≥ 5 score	KAA	1 = Qualified, 0 = Unqualified
Evaluation of information release and reporting on COVID-19	1 = ≤ 24 score, 2 = > 24 score	BAL	1 = Qualified, 0 = Unqualified
Evaluation of the effectiveness of government prevention and control of COVID-19	1 = ≤ 5 score, 2 = > 5 score	HRS	1 = Qualified, 0 = Unqualified
Appropriate behavior for self-control during COVID-19	1 = ≤ 2 score, 2 = > 2 score	SVH	1 = Qualified, 0 = Unqualified
Gender	1 = Male, 2 = Female	ID	1 = Qualified, 0 = Unqualified
Age	1 = 15–44,2 = 45–59, 3 = 60–69	CD	1 = Qualified, 0 = Unqualified
Eeducation level	1 = Primary School and below 2 = Junior high school 3 = Senior high schooland technical 4 = College and above	SAFA	1 = Qualified, 0 = Unqualified
Per capita annual income	1 = ≤ ¥5000, 2 = ¥5001-¥14,999 3 = ≥ ≥ ¥15,000	MC	1 = Qualified, 0 = Unqualified
Occupation	1 = Farmers, 2 = Others	HI	1 = Qualified, 0 = Unqualified
Marriage status	1 = In marriage, 2 = Others		

Firstly, analyzed the relationship between health literacy and COVID-19-related prevention and control KAP. As shown in Table 7, the availability of health literacy continued to be associated with knowledge, attitude, and practice for COVID-19 prevention and control after controlling for the population’s literacy and occupational factors. Compared with inadequate health literacy, those who with adequate health literacy had better knowledge of COVID-19 prevention and control [OR (95% CI) = 4.799 (3.861, 5.964)], more agreement on the responsibility of citizens to prevent and control infectious disease epidemics [OR (95% CI) = 4.862 (3.733, 6.333)], more recognition of COVID-19 related information reports evaluation [OR (95% CI) = 1.475 (1.197, 1.816)] and the effectiveness of government COVID-19 prevention and control [OR (95% CI) = 1.605 (1.233, 2.089)], and also more proactive in performing prevention and control behaviors [OR (95% CI) = 1.484 (1.208, 1.822)].

**Table 7:** Logistic regression analysis results of the relationship of meeting the overall standard of health literacy with COVID-19 prevention and control knowledge, attitude and practice.

Dependent variable	Independent variable	*b*	*S*_*b*_	*Wald χ*^2^	*P*	*OR* (95% *CI*)
Knowledge of prevention and control	Health literacy	1.57	0.11	199.97	< 0.001	4.80 (3.86, 5.96)
	Education level	0.15	0.06	7.74	0.005	1.17 (1.05, 1.30)
	Occupation	0.52	0.11	24.59	< 0.001	1.68 (1.37, 2.07)
	Constant	− 2.06	0.14	212.95	< 0.001	0.13
Responsibility of citizens to prevent and control the epidemic	Health literacy	1.58	0.14	137.57	< 0.001	4.86 (3.73, 6.33)
	Education level	0.20	0.05	14.19	< 0.001	1.22 (1.10, 1.36)
	Occupation	0.75	0.10	55.62	< 0.001	2.11 (1.74, 2.57)
	Constant	− 1.55	0.13	137.57	< 0.001	0.21
Evaluation of information release and reporting on COVID-19	Health literacy	0.39	0.11	13.36	< 0.001	1.48 (1.20, 1.82)
	Education level	0.16	0.05	9.43	0.002	1.18 (1.06, 1.30)
	Occupation	0.53	0.10	27.67	< 0.001	1.70 (1.40, 2.08)
	Per capita annual income	0.17	0.05	9.68	0.002	1.18 (1.06, 1.31)
	Constant	− 1.73	0.14	160.06	< 0.001	0.18
Evaluation of the effectiveness of government prevention and control of COVID-19	Health literacy	0.47	0.14	12.36	< 0.001	1.61 (1.23, 2.09)
	Marriage status	− 0.29	0.12	5.83	0.016	0.75 (0.60, 0.95)
	Education level	0.16	0.06	7.04	0.008	1.17 (1.04, 1.32)
	Occupation	0.30	0.11	7.14	0.008	1.35 (1.08, 1.68)
	Constant	0.60	0.19	10.08	0.001	1.81
Appropriate behavior for self-control during COVID-19	Health literacy	0.40	0.11	14.20	< 0.001	1.48 (1.21, 1.82)
	Education level	0.31	0.04	51.10	< 0.001	1.36 (1.24, 1.48)
	Constant	− 0.91	0.10	83.23	< 0.001	0.40

Secondly, analyzed the relationship between the three aspects of health literacy and COVID-19-related prevention and control KAP. Logistic regression was used to further analyze the relationship between the three aspects of health literacy and COVID-19-related prevention and control KAP. As shown in Table 8, the results showed that KAA and BAL were statistically significant with COVID-19 prevention and control knowledge, three aspects of prevention and control attitudes and prevention and control behaviors, while HRS was only shown to be unrelated to the government’s evaluation of prevention and control effectiveness and significantly associated with all the remaining prevention and control knowledge, attitudes and behaviors. And all residents with all three aspects of health literacy outperformed those without in terms of COVID-19 prevention and control KAP (all OR values > 1).

**Table 8:** Logistic regression analysis results of the relationship of the three aspects of health literacy with COVID-19-related prevention and control KAP.

Dependent variable	Independent variable	*b*	*S*_*b*_	*Wald χ*^2^	*P*	*OR* (95% *CI*)
Knowledge of prevention and control	KAA	1.56	0.10	228.68	< 0.001	4.78 (3.90, 5.85)
	BAL	1.51	0.11	201.96	< 0.001	4.54 (3.68, 5.59)
	HRS	1.38	0.11	155.10	< 0.001	3.96 (3.19, 4.92)
Responsibility of citizens to prevent and control the epidemic	KAA	1.65	0.13	173.90	< 0.001	5.19 (4.06, 6.63)
	BAL	1.62	0.13	153.86	< 0.001	5.05 (3.91, 6.53)
	HRS	1.34	0.13	106.98	< 0.001	3.80 (2.95, 4.89)
Evaluation of information release and reporting on COVID-19	KAA	0.35	0.10	12.62	< 0.001	1.42 (1.17, 1.73)
	BAL	0.52	0.10	25.65	< 0.001	1.69 (1.38, 2.06)
	HRS	0.57	0.11	27.43	< 0.001	1.76 (1.43, 2.18)
Evaluation of the effectiveness of government prevention and control of COVID-19	KAA	0.68	0.13	27.30	< 0.001	1.97 (1.53, 2.54)
	BAL	0.57	0.13	18.64	< 0.001	1.77 (1.37, 2.30)
	HRS*	–	–	–	–	–
Appropriate behavior for self-control during COVID-19	KAA	0.40	0.10	16.80	< 0.001	1.49 (1.23, 1.81)
	BAL	0.42	0.10	17.14	< 0.001	1.52 (1.25, 1.85)
	HRS	0.48	0.111	20.10	< 0.001	1.61 (1.31, 1.98)

*HRS was not associated with evaluation of the effectiveness of government prevention and control of COVID-19.

Thirdly, analyzed the relationship between the six dimensions of health literacy and COVID-19-related prevention and control KAP. Using the same method, we further analyzed the relationships between the six dimensions of health literacy and COVID-19-related prevention and control KAP. The results showed that the KAP of prevention and control of COVID-19 were statistically associated with SVH, SAFA, MC and HI, while the ID and CD were statistically associated with other contents of knowledge, belief and behavior except for the evaluation of COVID-19 related information reports. Similarly, residents with the six dimensions of health literacy had better knowledge of prevention and control, positive attitudes toward prevention and control, and proactive prevention and control appropriate behaviors than those who did not (all OR values > 1). The results were detailed in Table 9.

**Table 9:** Logistic regression analysis results of the relationship of the six dimensions of health literacy with COVID-19-related prevention and control KAP.

Dependent variable	Independent variable	*b*	*S*_*b*_	*Wald χ*^2^	*P*	*OR* (95% *CI*)
Knowledge of prevention and control	SVH	1.44	0.09	252.95	< 0.001	4.23 (3.54, 5.06)
	ID	0.99	0.10	103.43	< 0.001	2.70 (2.23, 3.26)
	CD	1.46	0.10	227.59	< 0.001	4.30 (3.56, 5.19)
	SAFA	1.64	0.09	322.16	< 0.001	5.15 (4.31, 6.17)
	MC	1.26	0.10	146.87	< 0.001	3.53 (2.48, 3.92)
	HI	1.23	0.10	167.23	< 0.001	3.41 (2.83, 4.11)
Responsibility of citizens to prevent and control the epidemic	SVH	1.76	0.10	294.92	< 0.001	5.79 (4.74, 7.07)
	ID	0.63	0.10	38.15	< 0.001	1.88 (1.54, 2.30)
	CD	1.83	0.12	236.63	< 0.001	6.24 (4.94, 7.88)
	SAFA	1.82	0.10	317.14	< 0.001	6.15 (5.04, 7.52)
	MC	1.14	0.12	95.30	< 0.001	3.12 (2.88, 4.33)
	HI	1.73	0.11	232.91	< 0.001	5.63 (4.51, 7.02)
Evaluation of information release and reporting on COVID-19	SVH	0.48	0.09	30.15	< 0.001	1.61 (1.36, 1.92)
	IDs*	–	–	–	–	–
	CD*	–	–	–	–	–
	SAFA	0.29	0.09	11.53	0.001	1.34 (1.13, 1.59)
	MC	0.35	0.10	11.76	0.001	1.42 (1.16, 1.73)
	HI	0.31	0.09	11.24	0.001	1.36 (1.14, 1.64)
Evaluation of the effectiveness of government prevention and control of COVID-19	SVH	0.46	0.10	19.72	< 0.001	1.59 (1.29, 1.95)
	ID	0.46	0.12	15.00	< 0.001	1.59 (1.26, 2.01)
	CD	0.55	0.12	22.16	< 0.001	1.73 (1.38, 2.18)
	SAFA	0.47	0.10	20.21	< 0.001	1.59 (1.30, 1.95)
	MC	0.42	0.13	10.94	0.001	1.52 (1.19, 1.95)
	HI	0.49	0.11	18.37	< 0.001	1.63 (1.30, 2.04)
Appropriate behavior for self-control during COVID-19	SVH	0.40	0.09	21.43	< 0.001	1.48 (1.26, 1.75)
	ID	0.43	0.10	20.26	< 0.001	1.53 (1.27, 1.85)
	CD	0.42	0.09	21.02	< 0.001	1.53 (1.27, 1.83)
	SAFA	0.53	0.09	39.63	< 0.001	1.71 (1.45, 2.02)
	MC	0.40	0.10	16.17	< 0.001	1.50 (1.23, 1.82)
	HI	0.29	0.09	9.73	0.002	1.33 (1.11, 1.59)

*No association with evaluation of information release and reporting on COVID-19.

## Discussion

COVID-19 is an acute respiratory infectious disease, and the transmission route of COVID-19 is specified as mainly via respiratory droplets and close contact^24^. Now that the local outbreak is well under control in China, the focus of prevention and control has shifted to continuing to guard against the dual risk of sporadic local dissemination of cases and imported cases from abroad. Early prevention is the key to control infectious diseases, and good health literacy of individual residents is an important guarantee to achieve early prevention^25,26^. Various efforts such as health education and health management have a positive contribution to the control of the COVID-19 epidemic, the mitigation of public panic and the orderly resumption of work, production and school^27,28^.

The results of this study showed that there is a close relationship between population health literacy and the KAP of COVID-19 epidemic prevention and control. Residents with adequate health literacy were more knowledgeable about COVID-19 epidemic prevention and control than those with inadequate health literacy; they were more likely to recognize the responsibility of citizens to prevent and control infectious disease epidemics; they were more likely to acknowledge the release and reporting of COVID-19-related information and the effectiveness of governmental efforts to prevent and control COVID-19 epidemics; and they were more likely to be proactive in taking actions that were feasible and appropriate during special times. In addition, compared with the association between sociodemographic characteristics such as gender, age and education level and KAP of COVID-19 epidemic prevention and control, whether residents have adequate health literacy is more closely related to KAP of COVID-19 epidemic prevention and control. Of the three health dimensions of health literacy, KAA and HRS were associated with each of the KAP of COVID-19 epidemic prevention and control survey components. Among the six health dimensions of health literacy, ID, AFA and HI were more closely associated with the KAP of COVID-19 epidemic prevention and control. Health literacy reflects a resident’s comprehensive ability to maintain their health and takes a longer time to develop^29^. The present status survey was completed in November 2020, less than 9 months after the COVID-19 epidemic began to spread globally, and it is unlikely that the health literacy level of each individual will change significantly in such a short period of time. Therefore, it can be inferred that there is an antecedent and consequential relationship between population health literacy and the KAP of COVID-19 epidemic prevention and control. The results of this study not only provide new evidence for understanding the importance of population health literacy, but also point out the focus of targeted health education to address the threat of sudden major infectious disease epidemics and fill the shortcomings of population health literacy.

In the “Health China 2030” plan, it is proposed that the national health literacy level (attainment rate) should be increased from 10% in 2015 to 20% in 2020, and then to 30% in 2030^30^. Residents’ health literacy, as an evaluation index to comprehensively reflect the development of national health, has been incorporated into the national health development plan^31^. According to this survey, the overall health literacy rate of residents in Shanxi Province, which belongs to North China, is 18.32%, which is higher than the health literacy level of 10.72%^32^ in Central China, and other investigations in South China^33,34^. However, great efforts are still needed to achieve the expected goal of national health literacy level. Health education is a way of communication in which individuals consciously create learning opportunities in order to improve health literacy^35^, and is a solid foundation for improving health literacy^36^, and the government and society should increase investment to integrate health education into everyone’s daily work, study, and life to form a good health literacy atmosphere and lay a solid foundation for resisting the threat of sudden major infectious disease outbreaks. The results of this study are only from a survey in Shanxi province, and the survey population is mainly rural residents, which cannot represent the national situation. In addition, the logistic regression analysis only controlled for the interference of socio-demographic factors involved in the health literacy survey, and there were other factors that could affect the relationship between health literacy and the KAP of COVID-19 epidemic prevention and control. Many previous studies have shown that the health literacy level of rural residents is significantly lower than that of urban residents^37,38^. The results of the logistic regression analysis in this study also showed that farmers had lower levels of the KAP of COVID-19 epidemic prevention and control than other occupational groups. The reason for this may be that the rural population is affected by a number of factors, including an underdeveloped economic level, poor basic life execution, poor health services, limited access to health information (e.g., primary care providers, specialists, blogs and magazines)^39^, and low awareness of adopting good health lifestyles and behaviors, which leads to a generally lower health literacy among rural residents, and consequently their the KAP of COVID-19 epidemic prevention and control levels are also lower.

## Conclusions

Overall, the health literacy rate of residents in Shanxi Province relatively was higher than other Provinces in Central and South China, and the residents’ health literacy was closely related to COVID-19 prevention and control KAP. Promoting residents’ health literacy by targeted health education can play an important and positive role in dealing with the threat of major infectious diseases outbreaks.

## Materials and methods

### Ethical statement

The study was conducted according to guidelines of the Declaration of Helsinki, and the National health literacy monitoring program of 2020 version issued by the National Health Commission. And this study was approved by the ethics committee of Chinese Center for Health Education, the participants signed informed consent form to participate in the study.

### Participants

This study was a cross-sectional survey and conducted in Shanxi, China, from September to November 2020. According to the requirements in the “Health literacy monitoring program for residents aged 15–69 in Shanxi Province in 2020”, the survey covers 10 counties and districts in Shanxi Province, with the same number of people assigned to the survey in each county. In all, 2700 citizens aged 15–69 years who had lived in the sampled regions for more than 6 of the previous 12 months were selected in Shanxi. A multistage stratified random sampling method^40^ was used to recruit participants. The sampling method was divided into four stages. Firstly, 3 randomly selected townships (streets) in each of the 10 counties and districts in Shanxi Province using the systematic sampling method ranked by population size. Secondly, 2 administrative villages (neighbourhood committees) were randomly selected from each township (street) using the systematic sample ranked by population size. Thirdly, within each sampled administrative village (neighbourhood committee), residential households were divided into villagers/resident groups on a scale of 40–60 households and one villager/resident group was selected by the simple random sampling method. Finally, one permanent resident aged 15–69 years old was selected from the sampled households according to the KISH table method.

### Questionnaire

Data were obtained by using the National health literacy monitoring questionnaire for 2020 issued by the Chinese Center for Health Education. The questionnaire consisted of three parts: (1) basic personal situation, (2) health literacy content, and (3) knowledge, attitude, practice for prevention and control of COVID-19 questionnaire. Based on the “Chinese Resident Health Literacy—Basic Knowledge and Skills (Trial)” and existing public health issues in China, the health literacy Sect. (56 questions) was further categorized into three aspects and six dimensions, total score of 73 points. The three aspects were (1) knowledge and attitudes (KAA), (2) health-related behaviour and lifestyle (BAL), and (3) health-related skills (HRS). The six dimensions were (1) scientific views of health (SVH), (2) infectious diseases (ID), (3) chronic diseases (CD), (4) safety and first aid (SAFA), (5) medical care (MC), and (6) health information (HI). An overall health literacy score was computed as the sum of all three aspects and six dimensions. The participants were divided into 2 categories: (1) people with inadequate health literacy (total health literacy score < 80% of the overall score, with a total score < 58 points) and (2) people with adequate health literacy (total health literacy score ≥ 80% of the overall score, with a total score ≥ 58 points).

The KAP for prevention and control of COVID-19 questionnaire covers the knowledge of prevention and control (11 items, single- or multiple-choice questions with “don’t know” option), the responsibility for the prevention and control of infectious disease transmission (6 items, yes/no multiple-choice questions), the evaluation for COVID-19-related information release and reporting (5 items, five-level single-choice questions), the evaluation for the government’s COVID-19 prevention and control results (one item, five-level single-choice question), and the practice concerning appropriate self-prevention and control behaviors during the COVID-19 outbreak (7 items, yes/no multiple-choice questions).

### Site investigation and quality control

In this study, the on-site survey was conducted by means of a household survey, and the respondents were encouraged to complete the questionnaire by themselves. If the respondents could not complete the questionnaire independently, the questionnaire was completed by a uniformly trained and qualified surveyor in the form of face-to-face questioning. Quality control is performed using a three-stage quality control method before, during and after the survey. Before the survey, the Shanxi Center for Health Education unified to complete the extraction and coding of households and the training of investigators. In the survey, the counties and districts use a uniformly printed questionnaire, the investigator does not use inducing or suggestive language, review the completion of the questionnaire on the spot, check for gaps, and finally fill in the name of the investigator and other survey completion information. After the survey, the quality control officers in each county and district to review the township/street questionnaires in a timely manner, each township/street to take 15 questionnaires, if more than 3 unqualified questionnaires, the township/street site survey work is considered unqualified, must be re-surveyed.

### Statistical analysis

EpiData 3.0 software was used to establish the database, and data were double-entered for verification; statistical analysis was performed using SPSS 25.0 (SPSS, Inc., Chicago, IL, USA). COVID-19 prevention and control knowledge score: 1 point for correct answers, 0 points for others; 11 points in total. Prevention and control attitude, prevention and control evaluation score: 1 point for “yes”, 0 points for “no” in multiple-choice questions; five levels of evaluation questions: 1 point for “disagree/poor”, 2 points for “agree/poor”, 3 points for “average”, 4 points for “agree/good”, 5 points for “strongly agree/very good”; prevention and control behavior: 1 point for “measures”, 0 points for “no measures”, 7 points in total. The median scores of COVID-19 prevention and control knowledge, attitude, evaluation, and behavior were calculated, and the respondents were divided into “good” and “poor” knowledge of prevention and control, “positive” and “negative” attitude of prevention and control, and “active” and “passive” behavior of prevention and control, which were used as dependent variables respectively. The groups were divided into 2 groups according to whether they had health literacy or not (inadequate health literacy group and adequate health literacy group), and the rates or composition ratios of COVID-19 prevention and control knowledge, attitude, valuation, and behavior in the 2 groups were compared using Chi-square test or Wilcoxon rank sum test. Controlling for the confounding effect of 2 groups of sociodemographic characteristics, multifactorial binary logistic regression analysis by stepwise method (α in = 0.05, α out = 0.1) was used to analyze the relationship between health literacy, three aspects and six dimensions of health literacy and the scores of COVID-19 prevention and control knowledge, attitude, evaluation, and behavior. *P* < 0.05 was considered statistically significant.

### Consent to publish

All the authors consent to publish the article in its present form.

## Data Availability

The data supporting the conclusions of this article are included within the article.

## Abbreviations

KAP: Knowledge, attitude and practice
KAA: Knowledge and attitudes
BAL: Behaviour and lifestyle
HRS: Health-related skills
SVH: Scientific views of health
ID: Infectious diseases
CD: Chronic diseases
SAFA: Safety and first aid
MC: Medical care
HI: Health information
